# Physiological Mechanisms Only Tell Half Story: Multiple Biological Processes are involved in Regulating Freezing Tolerance of Imbibed *Lactuca sativa* Seeds

**DOI:** 10.1038/srep44166

**Published:** 2017-03-13

**Authors:** Ganesh K. Jaganathan, Yingying Han, Weijie Li, Danping Song, Xiaoyan Song, Mengqi Shen, Qiang Zhou, Chenxue Zhang, Baolin Liu

**Affiliations:** 1 Institute of Biothermal Science and Technology, University of Shanghai for Science and technology, 516 Jungong Road, Shanghai 200093, China

## Abstract

The physiological mechanisms by which imbibed seeds survive freezing temperatures in their natural environment have been categorized as freezing avoidance by supercooling and freezing tolerance by extracellular freeze-desiccation, but the biochemical and molecular mechanisms conferring seed freezing tolerance is unexplored. In this study, using imbibed *Lactuca sativa* seeds we show that fast cooled seeds (60 °C h^−1^) suffered significantly higher membrane damage at temperature between −20 °C and −10 °C than slow cooled (3 °Ch^−1^) seeds (*P* < 0.05), presumably explaining viability loss during fast cooling when temperature approaches −20 °C. Total soluble sugars increase in low temperature environment, but did not differ significantly between two cooling rates (*P* > 0.05). However, both SOD activity and accumulation of free proline were induced significantly after slow cooling to −20 °C compared with fast cooling. RNA-seq demonstrated that multiple pathways were differentially regulated between slow and fast cooling. Real-time verification of some differentially expressed genes (DEGs) revealed that fast cooling caused mRNA level changes of plant hormone and ubiquitionation pathways at higher sub-zero temperature, whilst slow cooling caused mRNA level change of those pathways at lower sub-zero ttemperatures. Thus, we conclude that imbibed seed tolerate low temperature not only by physiological mechanisms but also by biochemical and molecular changes.

Temperature is a major environmental sieve selecting plant distribution in temperate and cold climates^1^. In many complex environments, plants have to cope with temperatures plummeting as low as −30 °C during winter^2^. However, most species adapted to these locations have evolved with a suite of protective mechanisms, which are mostly triggered during a brief exposure to low but non-freezing temperature, in light of preparing the plants to further lower temperature, a process termed as ‘cold acclimation’^3^. Understanding the mechanisms of freezing tolerance in plants is of extensive ecological and practical importance. Not only do such studies inform us about the coping strategies plants use to survive freezing in natural conditions, but they also offer great insights to improve the freezing tolerance of many agronomic plants that are susceptible to freezing damage.

Research into identifying the mechanisms by which plants survive low temperature has established a multitude of molecular, biochemical, and physiological changes are involved in making a plant freezing tolerant. One premise is that an array of genes is either up- or down-regulated in response to low temperature^4^,^5^,^6^. These waves of changes at the transcript levels expressing or suppressing the genes presumably encompass numerous functions including transcription, signalling, cell wall biogenesis, and synthesis of hormones^4^. Another line of evidence shows that numerous phytohormones including abscisic acid (ABA), auxin, ethylene and cytokinin play a crucial role in regulating freezing tolerance processes. Whereas the level of ABA in plants increases in the low temperature environment^7^, auxin^8^, cytokinin and ethylene^9^ are mostly down regulated in many species. These changes in phytohormone levels alter gene expression by inducing or preventing the degradation of transcriptional regulators via the ubiquitin-proteasome system^10^.

In addition, the levels of soluble carbohydrate in many plant tissues are known to increase during low temperature^11^. These compounds are proposed to serve as cryoprotectants and prevent the freezable water present in cells undergoing a phase change^12^. Generally, water present in the biological samples forms ice when exposed to low temperature and more than the traceable quantity of ice in the intracellular region is mostly lethal. However, plants mitigate this stress by either holding the water in a supercooled state or limiting the freezing to extracellular spaces and prevent intracellular ice formation (IIF)^13^. Thus, successful survival of plant organs depends invariably on the ability to avoid IIF.

One of the widely held assumptions is that plants adapted to a cold-environment escape the mortality associated risks of a generative phase by completing the reproduction in summer and shed the seeds before the onset of winter. These seeds subsequently establish soil seed banks and over-winter under dense snow cover before beginning to germinate the next summer^14^,^15^. Because snow-cover provides a benign environment to the over-wintering seeds, the risk of mortality resulting from ice formation as a consequence of low temperature is minimized^16^. Therefore, significant attention has been paid to understand the survival strategies of individual plant parts overwintering above the snow cover, e.g. buds, bark or leaves^17^,^18^. Thus, information on seed survival strategies in a low temperature environment is scant, despite some interest in understanding the tolerance mechanisms of seedlings, mainly due to the assertion that newly germinated seedlings are susceptible to freezing injury in exposed soil surfaces. However, at the time of snow-melt in late winter, seeds in the soil seed banks are hydrated by the melting snow and subsequently exposed to nocturnal temperature plummeting to sub-zero levels. Such events are expected to occur more commonly in the future due to the effects of global warming reducing the duration of snow cover, thereby leaving the seeds exposed to direct low temperature late in winter.

The physiological mechanisms, by which the seeds survive freezing stress have been studied in a few species including *Arabidopsis thaliana*^19^, *Brassica napus* cv. Quest^20^, Eurotia *lanata* (Pursh) Moq^21^, *Lactuca sativa*^22^,^23^,^24^, *Pinus sylvestris*^25^ and *Pyrus malus*^26^,^27^. These studies show that imbibed seeds survive low temperature either by freezing avoidance by supercooling or freezing tolerance by extracellular-freezing. In *Lactuca sativa*, there is evidence to show that the selection of a particular mechanism is related to the rate at which seeds are cooled. At an environmental cooling rate of <4 °C h^−1^, the seeds survived low temperature by freezing tolerance, whereas at faster cooling rates the seeds undergo supercooling^23^. However, the putative biochemical and molecular changes in seeds during low temperature survival in the imbibed state remain largely superficial, promoting the need for further investigation.

Here we investigate the biochemical and molecular changes that occur in imbibed seeds when exposed to low temperature. *Lactuca sativa* has been extensively used as a model species to advance our understanding on the physiological mechanisms operating in imbibed seeds^22^,^23^,^24^. Therefore, we believe this species might be a better candidate for identifying the biochemical and molecular mechanisms. Our specific objectives in this study with imbibed *L. sativa* seeds were (1) to revisit the physiological mechanisms at different cooling rates; (2) to characterize the biochemical change of imbibed freezing seeds at empirical and ecologically meaningful cooling rates; and (3) to determine the differential molecular changes occurring in seeds under different cooling rates.

## Results

### Seed imbibition and germination

**Figure 1:**
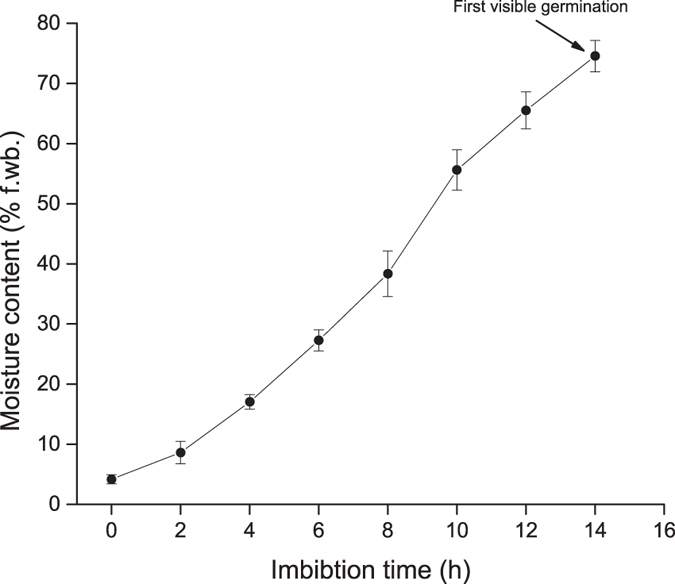
Percentage of water imbibed by *Lactuca sativa* cv. Iceberg seeds when imbibed above moist filter paper in 9 mm diameter Petri plates. Seeds at 0 h are from 15% RH and 15 °C.

The rate at which moisture of the L. sativa seeds incubated at 21 °C increased is shown in Fig. 1. The pattern of water absorption is tri-phasic. The seeds absorb water rapidly when they come in contact with water. However, this rapid uptake of water only lasts for some time (from few minutes to few hours depending upon the species). This is usually followed by a slow water uptake phase, loosely termed as lag-phase, where the seeds do not absorb much water, rather use this time to move the hydrated reserved and begin the reactions at molecular levels that require water. Once this phase is completed the radicle begins to emerge, where additional water is absorbed. Seeds of lettuce used in this study began to germinate after 14 h and therefore seeds residing above agar at 21 °C for 12 h were considered as fully imbibed (Fig. 1). One hundred percent of the seeds germinated within 5 days, indicating the high viability of the seed lot.

### Freezing treatment and viability assessment

**Figure 2:**
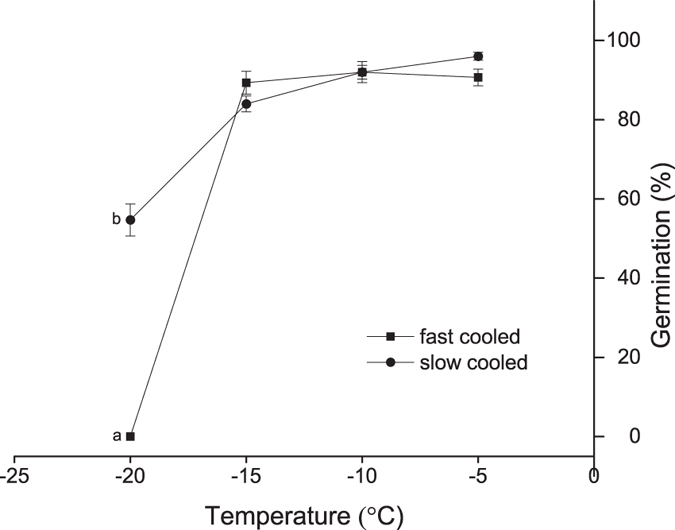
Germination percentage of *Lactuca sativa* seeds following cooling at 60 °Ch^−1^ (fast cooled) and 3 °Ch^−1^ (slow cooled) to various sub-zero temperatures. No significant difference in germination percentage was observed until the temperature dropped to −15 °C at both cooling rate. Seeds cooled to −20 °C at a faster rate showed no survival but slow cooled seeds resulted in significantly higher germination percentage (indicated with lower-case alphabet).

Germination of fully imbibed seeds declined with decreasing temperature. Seeds cooled at both cooling rates survived to a higher percentage above −15 °C (Fig. 2). However, none of the fast cooled seeds survived at −20 °C, compared with 54% survival of the slowly cooled seeds that germinated to normal seedlings when retrieved at −20 °C (Fig. 2).

### Electrolyte leakage analysis

**Figure 3:**
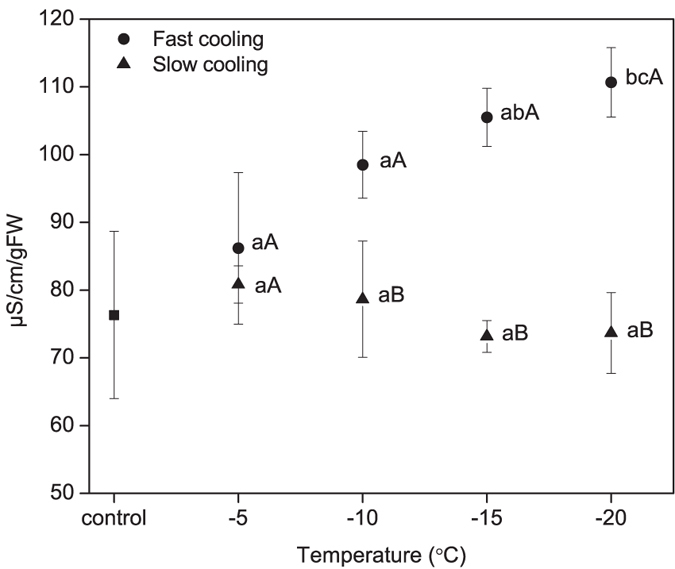
The electrolyte leakage analysis of imbibed seeds without freezing (control) and after cooled −20 °C at 3 °Ch^−1^ (slow cooling) or 60 °Ch^−1^ (fast cooling). Different upper case letters indicate statistically significant difference between two different cooling rates at a particular low-temperature. Different lower case letters indicate significant difference at different low temperatures.

No significant difference in electrolyte leakage was found between control and seeds cooled at either cooling rate above −15 °C (*P* > 0.05; Fig. 3). However, seeds cooled at the faster rate to −20 °C showed significantly higher leakage than control seeds and slow cooled seeds (*P* < 0.05). The difference in electrolyte leakage between fast and slow cooled seeds became apparent as high as −10 °C (Fig. 3). Overall, the results indicate that fast cooled seeds suffered significantly higher membrane damage at temperatures between −15 and −20 °C (Fig. 3).

### Superoxide Dismutase (SOD) activity was upregulated by slow cooling treatment at −20 °C

**Figure 4:**
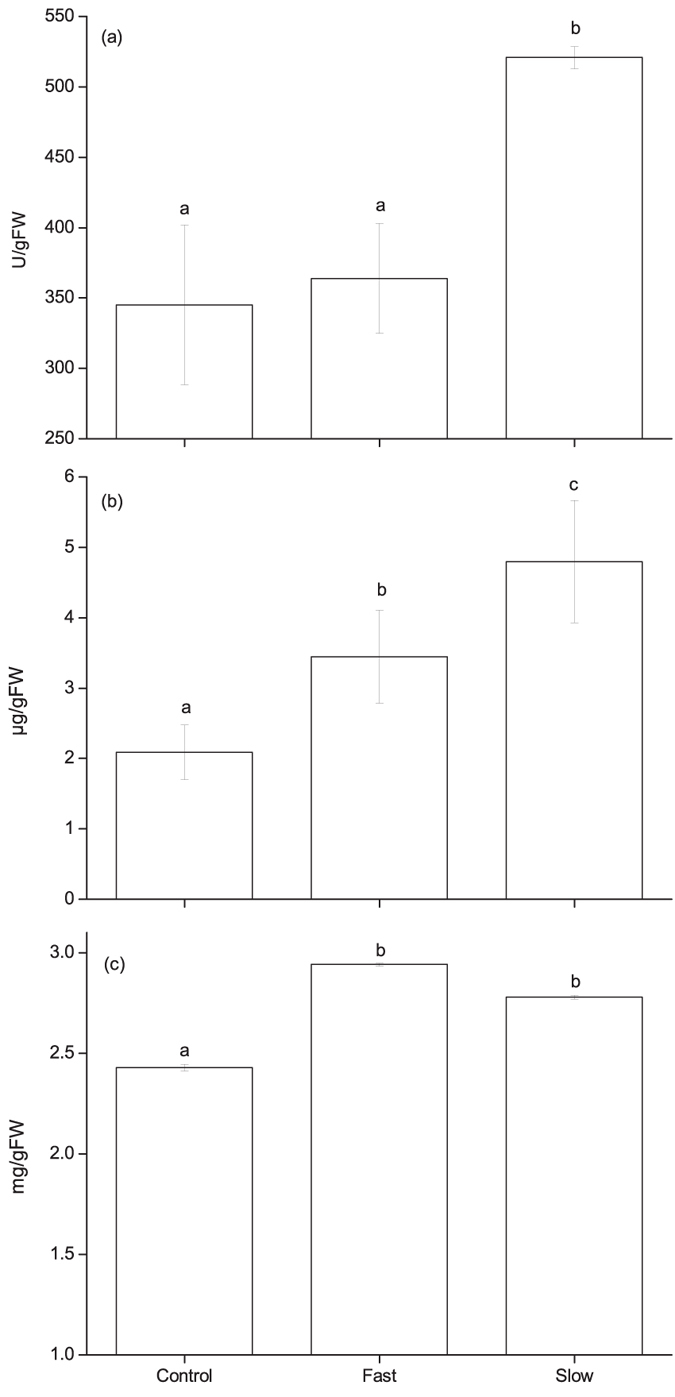
The physiological changes in process of programmed cooling treatment with fast and slow cooling rates. (**a**) SOD activity in seeds imbibed for 12 hours without freezing (control) and after cooled to −20 °C at 3 °Ch^−1^ (slow cooling) or 60 °Ch^−1^ (fast cooling). (**b**) The relative accumulation level of free proline in seeds imbibed for 12 hours without freezing (control) and after cooled to −20 °C at 3 °Ch^−1^ (slow cooling) or 60 °Ch^−1^ (fast cooling). (**c**) Soluble sugar content measured in seeds imbibed for 12 hours without freezing (control) and after cooled to −20 °C at 3 °Ch^−1^ (slow cooling) or 60 °Ch^−1^ (fast cooling). Statistically significant difference between groups are marked with different alphabets.

Imbibed seeds slowly cooled to −20 °C had significantly higher SOD activity (521.60 U/g) than both control (355.54 U/g) and fast cooled samples (360.15 U/g) (Fig. 4a). However, SOD activity difference was not significant between fast cooled seeds retrieved at −20 °C and control (*P* > 0.05).

### Slow cooling caused higher accumulation of free proline at −20 °C

The free proline based on UV absorbance in seeds fast cooled to −20 °C did not differ significantly to the seeds prior to cooling (i.e. control) (3.59 ug/g FW vs 2.16 ug/g FW, *P* > 0.05, Fig. 4b). But a significant induction of free proline was observed in the seeds cooled slowly to −20 °C (4.60 ug/g FW) when compared with control seeds (*P* < 0.05) (Fig. 4b).

### Soluble sugar content is not related to the freezing tolerance developed in slow cooling treatment

The level of soluble sugar was increased in seeds exposed to low temperature compared with control (Fig. 4c). Although the seeds cooled at 60 °Ch^−1^ resulted in more levels of sucrose (2.93 mg/g FW) compared with seeds cooled at 3 °C h^−1^ (2.77 mg/g FW), there was no statistically significant difference of sucrose content recorded between two cooling rates (*P* > 0.05).

### Auxin and ABA expressed significantly higher in slow cooled seeds

Under control condition, the content of ABA was 0.159 ± 0.078 ug g^−1^ FW, and the IAA content was 0.125 ± 0.012 ug g^−1^ FW. However, both fast and slow cooling led to significantly higher accumulation of ABA and IAA (*P* < 0.05). In particular, seeds cooled slowly to −20 °C resulted in significantly higher level of ABA (1.033 ± 0.043 ug g^−1^ FW for fast cooling vs. 1.406 ± 0.13 ug g^−1^ FW for slow cooling; *P* < 0.05) and IAA (0.256 ± 0.015 ug g^−1^ FW for fast cooling vs. 0.477 ± 0.027 ug g^−1^ FW for slow cooling, *P* < 0.05).

### RNA-seq analysis

RNA-seq analysis conducted on fully imbibed seeds cooled to −20 °C at two different cooling rates showed that an average of 12,000,000 reads were obtained in each sample (Table 1). We were able to map 65~67% of the total reads to the genome sequences with more than 65% of the total reads uniquely matching and the multi-position matched reads occupied no more than 0.67% (Table 1).

**Figure 5:**
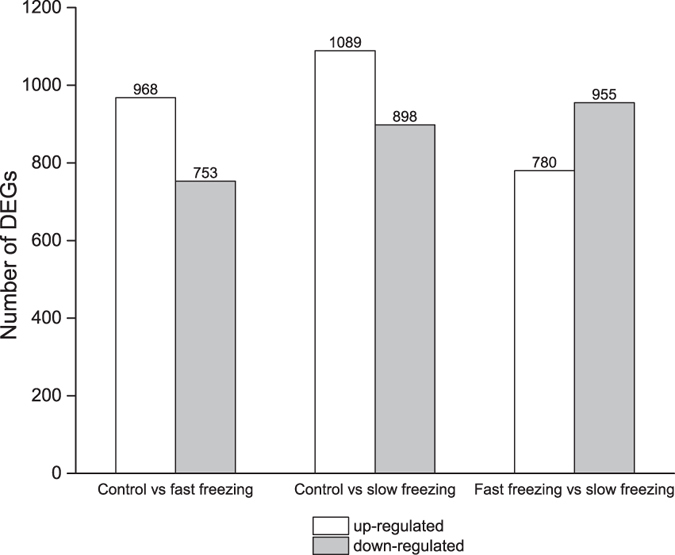
Number of differentially expressed genes (DEGs) amongst control, fast and slow cooling treatment.

The number of differentially expressed genes (DEGs) varied significantly between the treatments (Fig. 5). There were 1721 DEGs between control and fast cooling treatment, in which 968 were upregulated and 753 were down-regulated. A total of 1987 DEGs were observed between control and slow cooling treatment in which 1089 were upregulated and 898 were down-regulated. However, out of 1735 DEGs between fast and slow cooling treatment, 780 were identified to be upregulated and 955 were down-regulated. Notably, there were 570 DEGs in both fast and slow cooling when compared with control treatment, indicating these were cold responsive genes. Of these 570 DEGs, 11 genes including leucine-rich repeat extensin-like protein 4-like and clock regulator Time For Coffee, *etc* were up-regulated during fast cooling but down-regulated during slow cooling. By contrast, lanC-like protein-2 like down-regulated during fast cooling was up-regulated when seeds were cooled slowly. All the 12 genes expressed in opposite directions between fast and slow cooling are listed in Table 2.

### Pathway enrichment

There were 1027 DEGs that can be annotated to KEGG pathways between control and slow cooling treatment; 904 between control and fast cooling treatment; 789 between fast and slow cooling treatment. Based on the pathway enrichment results using DEGs, the significant pathways between control, fast and slow cooled treatments can be classified into 4 types (Table 3). Type I is the pathways that differed significantly only in slow cooling based on the comparison with control, and include RNA transport, spliceosome, plant hormone signal, endocytosis, pyrimidine metabolism, and nucleotide excision repair. Type II encompasses pathways that were induced in both cooling treatments but enriched more significantly in slow cooling treatment than fast cooling, and include homologous recombination, DNA replication, and ubiquitin mediated proteolysis. Type III contains pathways that differed significantly only in fast cooling based on the comparison with control and include alanine, aspartate and glutamate metabolism and fatty acid elongation. Type IV contains pathways that were induced with no difference between the two cooling rates. This type includes protein processing in the endoplasmic reticulum and plant circadian rhythm. The pathways included in type IV may be the common pathways of both fast and slow cooling treatment.

### Verification of factors in plant hormone and ubiquitin signal pathways by real time PCR

**Figure 6:**
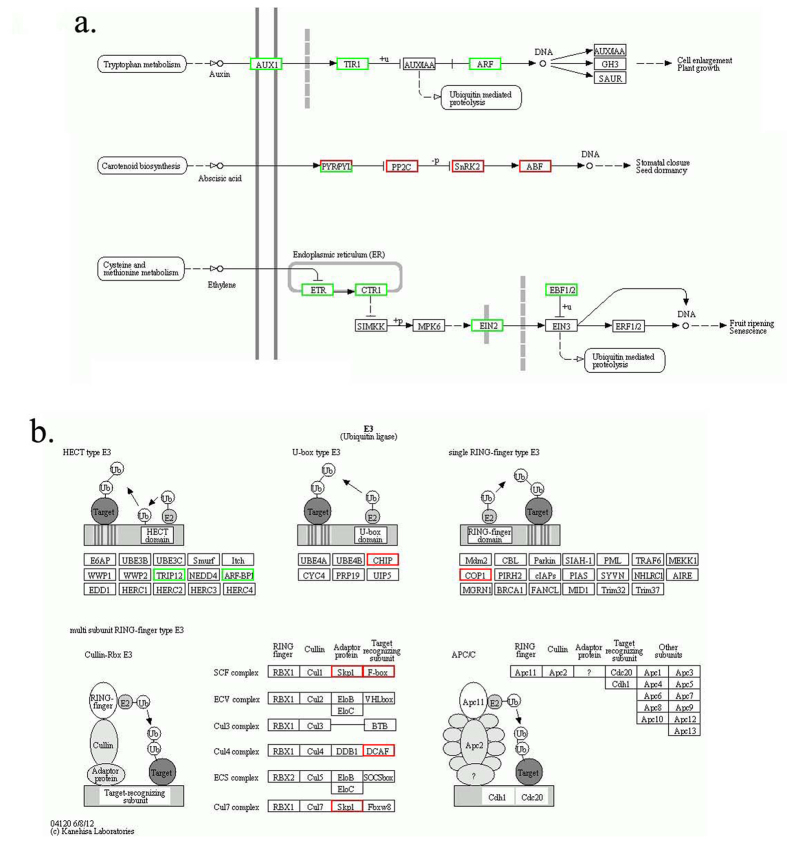
The mRNA level change of factors in plant hormone pathway (**A**) and ubiquitinn E3 ligase (**B**) between fast and slow freezing based on RNA-seq^59^. The red rectangles represent up-regulation and green rectangles represent down-regulation of genes in slow freezing compared with fast freezing.

Pathway enrichment results indicated that both plant hormone and ubiquitin E3 ligase mediated proteolysis were modified during slow cooling treatment compared with fast cooled seeds (Fig. 6). According to RNA-seq, after cooling the imbibed seeds at 3 °Ch^−1^ to −20 °C, the transcription of ARF1 was induced, while TIR1 was reduced in fast-cooled seeds. In addition, the transcription of ethylene pathway factors including EBF and EIN2 were reduced, but the ABA pathway gene ABF was induced at −20 °C (Fig. 6).

**Figure 7:**
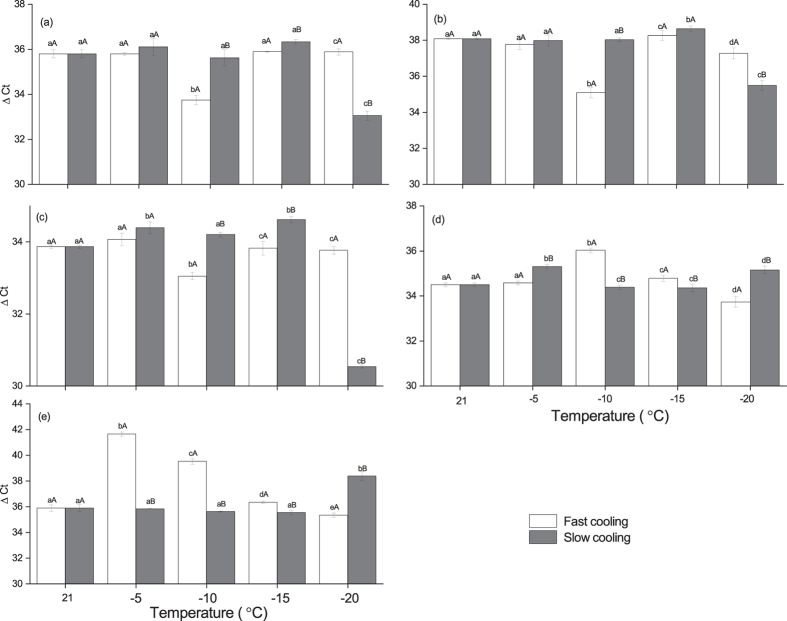
The verification of RNA-seq for genes in plant hormone pathways by real time PCR, (**a**) EIN2; (**b**) EBF; (**c**) TIR1; (**d**) ARF1 and (**e**) ABF. Different lower case letters indicate the significant difference between temperatures. Different higher case letters indicate the significant difference between cooling rates.

**Figure 8:**
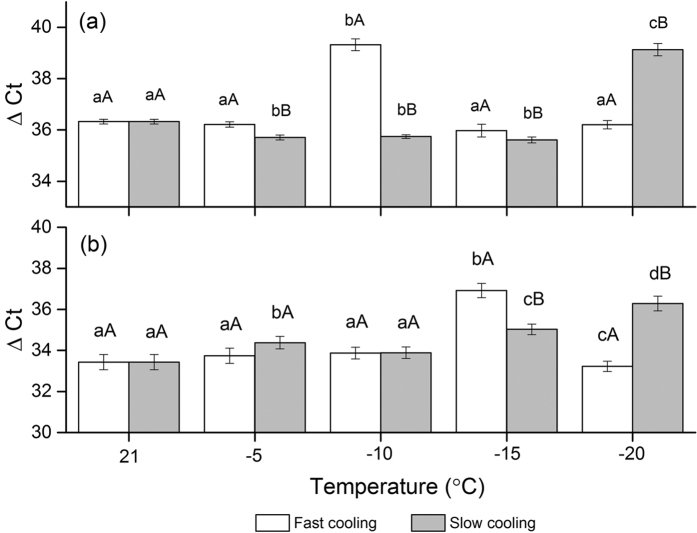
Verification of RNA-seq for factors in SCF complex by real time PCR, (**a**) F-box and (**b**) SKP 1. Different lower case letters indicate the significant difference between temperatures. Different higher case letters indicate the significant difference between cooling rates.

We performed real-time PCR to specifically characterize the temperature at which the mRNA level changes for factors of these two pathways by determining the changes after cooling the seeds to −5, −10, −15 and −20 °C (Figs 7 and 8). During slow cooling, the mRNA level change of above mentioned factors occurred at −20 °C, and no significant change was evident at −5, −10 and −15 °C (Fig. 7). However, during fast cooling treatment, although there was no significant mRNA level change for above plant hormone pathway factors at −20 °C, the mRNA level change of auxin and ethylene pathway related factors including ARF1, TIR1, EIN2, EBF occurred at temperatures as above at −10 °C. Nevertheless, this increased mRNA level went back to original level at −15 °C at fast cooling treatment (Fig. 7, Table 4). In contrast, the mRNA level change of ABF occurred at −5 °C during fast cooling which indicates the faster response of ABA to cooling than auxin and ethylene signals (Table 4). However, the mRNA level of above factors gradually went back to the original level in the seeds cooled to −15 °C at a fast rate. Although the temperature at which the mRNA level changed varied between cooling rates, the tendency to increase or decrease was the same at both cooling rates. For example, ARF1 and ABF showed positive response while TIR1, EIN2 and EBF showed negative response under both cooling treatments (Fig. 7, Table 4). Moreover, the two auxin-related factors exhibited antagonistic expression in seeds, while EIN2 and EBF exhibited synergistic expression (Fig. 7, Table 4).

The ubiquitin mediated proteolysis pathway (enriched) was significantly different between slow cooling and fast cooling treatments (Table 3). Since SCF has been proven to be involved in plant hormone signal pathways, we detected the duration change of SCF factors including Skp1 and F-BOX (Fig. 8). Both Skp1 and F-BOX were induced by slow cooling treatment according to RNA-seq. The real time PCR verification was consistent with the RNA-seq results (Fig. 6). The peak level of Skp1 existed at −15 °C during fast cooling and −20 °C when the seeds were cooled at the slower rate of 3 °Ch^−1^. The peak level of F-BOX existed at −10 °C and −20 °C in fast and slow cooled seeds, respectively. Similar to the factors of plant hormone signal pathway in fast cooling treatment, the expression levels of these factors went back to the original level at later stage(s) (Fig. 8, Table 4).

## Discussion

A combination of physiological and molecular events occurred in imbibed *L. sativa* seeds when exposed to low temperature. Based upon freezing test results, it appears that iceberg lettuce seeds undergo supercooling at the faster cooling rate, but at a slower or more ecologically meaningful cooling rate the seeds develop freezing tolerance by extracellular freeze induced desiccation as a survival mechanism contributing to survival below −15 °C (Fig. 1). This finding is in accordance with the previous results on several cultivars of lettuce as proposed by Keefe and Moore^23^. However, this pattern of survival strategy is not universal in seeds because some cultivators of lettuce did not survive −20 °C regardless of cooling rate^24^,^28^. Such discrepancy is also found in other seed systems tested, e.g. *Pinus sylvestris*^25^. Furthermore, the successful survival of iceberg lettuce seeds by extracellular freezing has a lower limit of around −40 °C compared with supercooled seeds losing viability at temperatures around −18 °C^29^.

From the premise that membrane damage is directly related to the morality of seeds at low temperature^30^, the results that slow cooled seeds show relatively little or no membrane damage compared with fast cooled seeds when cooled to −20 °C are of particular interest. The increased electrolyte leakage at −20 °C during fast cooling corroborate with the mortality of seeds at this temperature suggesting that membrane damage occurred possibly due to the formation of ice in supercooled water disrupting the membranes. However, various biochemical substances produced in seeds are known to protect seeds under low temperature environment. Soluble sugars are present in seeds of many species have been proposed to play an important role in conferring seed survival during stress related to desiccation. When the free water present in the seeds is removed, the sugars actively participate in maintaining the structural integrity of cells and preventing the disruption of cellular structures at a wide-range of temperature up to −196 °C. In some woody plants, soluble sugars accumulate from autumn to winter which correlated with an increase in freezing tolerance^31^. Further, it has been reported that cold-acclimation results in increased soluble sugar levels, specifically raffinose and stachyose, which would subsequently increase the freezing tolerance capacity of many plant parts^32^. However, whether the levels of soluble sugar affect the extent of freezing tolerance in seeds could not be determined due to the small increase in soluble sugar at both cooling rate (Fig. 3). Alternatively, imbibition in seeds may trigger germination events which might increase the soluble sugar levels. Thus, the importance of soluble sugars in freezing tolerance of seeds still awaits more specified investigation.

Nevertheless, the increased amount of SOD activity and proline accumulation during slow cooling indicate these changes likely play a pivotal role in protecting membranes during freezing. The damage of membrane integrity at a low temperature environment is attributed to the phase transition of membrane fatty acids from liquid-crystalline to solid-gel state due to the degradation of lipids by enzymes^33^. The second set of explanation for membrane damage during low temperature is the stress environment produces free radical and reactive oxygen species (ROS) which affect cellular functions^34^. Many studies have proposed that SOD will reduce ROS, thus protect membrane during low temperature stress^35^. Emerging findings also support the importance of free proline in protecting membranes^36^,^37^. Results of this study strongly suggest that slow cooling induces higher proline accumulation and SOD activity to maintain membrane integrity.

In the recent years, a great deal of attention has been given to identify the genes involved in plant freezing tolerance. In recent years, RNA-seq is becoming a revolutionary tool for investigating the molecular mechanism in a robust way^38^ and this method has been successfully applied for seed systems^39^. In the present study, we used RNA-seq to determine the changes of gene expressions occurring in seeds when cooled to −20 °C. In line with the current understanding of plants and seedlings, our results show that numerous genes and functional pathways were up or down-regulated in imbibed lettuce seeds when moved to low temperature conditions from room temperature (Fig. 5).

Approximately 550 genes changed during both fast and slow cooling indicating that these genes were expressed as a result of low temperature environment. However, 12 of these genes were either up-regulated or down-regulated depending on the cooling rate employed. In this regard, it is of interest to note that leucine-rich repeat extensin-like protein 4-like, clock regulator Time For Coffee and a dead-box RNA helicase up-regulated during fast cooling treatment were down-regulated when seeds were cooled slowly. Leucine-rich repeat extensin-like protein 4 is a protein related to root morphogenesis and pollen development^40^, as well as the plant -microbe interaction^41^. However, there has been no report about the correlation of this gene with low temperature. The TIME FOR COFFEE (TIC) encodes a nuclear regulator in the *Arabidopsis thaliana* circadian clocks^42^. TIC acts as a negative factor in the JA-signaling pathway which mediates various stress responses^43^. This is the first study showing the involvement of this gene in cold tolerance, but the interaction of circadian rhythm and cold acclimation has been reported in voluminous studies^44^. Likewise, One DEAD-box ATP-dependent RNA helicase was found to be up- and down-regulated during fast and slow cooling respectively. The DEAD-box helicase is believed to play an important role in cold stress defense in bacteria by the unwinding cold-stabilized secondary structure in the 5′-UTR of RNA^45^. On the other hand, lanC-like protein 2 down-regulated during fast cooling was up-regulated during slow cooling. LanC-like protein 2 is similar to G protein coupled receptor (GCR) 2-like genes. GCR 2 is identified to be the membrane receptor of ABA^46^. In lettuce, LanC may be the key receptor gene in ABA signal during low temperature stress^47^.

Our study also focused on relating these genes to functional pathways occurring in imbibed seeds after cooling the seeds at two different cooling rates. Based on pathway enrichment of DEGs of control-fast cooling treatment, control-slow cooling treatment and fast-slow cooling treatment, the significant pathways between fast and slow cooling treatment at −20 °C can be classified into 4 types, which included pathways enriched in slow cooling or enriched in fast cooling treatment, or both (Table 3). It seems that the regulation of genetic or epigenetic aspects was changed more after slow cooling treatment such as RNA transport, DNA replication and ubiquitin mediated proteolysis, while metabolism of small chemicals including alanine, aspartate, glutamate metabolism and fatty acid elongation were induced more during fast cooling. There are reports about the cold response of RNA processing including RNA splicing, export and secondary structure unwinding in plants^48^ and the induction of Ala, Asp and Glu under cold stress^49^. However, it remains unknown at this time, whether sudden temperature plummetleads to cytoplasmic biochemical change at lower sub-zero temperature while the slow cooling leads to gene expression change in the nucleus.at the same point.

Pathway enrichment based on RNA-seq indicated that plant hormones including ABA, auxin, and ethylene are believed to be an important regulator of cold stress response^8^. Ubiquitination also plays a significant role in cold acclimation^50^. The two factors in auxin pathway including ARF1 and TIR1 are the targets of miR393 which is involved in cold stress^51^. Ethylene pathway factors EBF and EIN2 are known to involve in fruit ripening disorder caused by chilling injury^52^. ABF is an important regulator in ABA response pathway. ABF/ABA-responsive element binding protein (AREB) are basic region leucine zipper (bZIP)-type DNA-binding proteins that regulate the expression of Cor (Cold Responsive)/Lea genes that contain cis-acting elements ABA-responsive element (ABREs) in their promoters^53^. F-box and Skp1 are components of E3 ligase. However, there has been no report about the involvement of Skp1 and F-box in cold response. Nevertheless, based on our RNA-seq results, Skp1 and F-box were up-regulated at −20 °C after slow freezing. How Skp1 and F-box are involved in freezing tolerance of seeds remains an open area to be investigated in the future.

To date, most of the freezing tolerance studies on physiological and molecular mechanisms involved in plants have focused on plant tissues and there have been no in-depth studies available on seed systems (see Introduction). Whilst, it is clear that the mechanisms involved in plants vary with species or plant parts under investigation, several new pathways that are identified here to involve in seed freezing tolerance^1^,^2^,^3^. A direct comparison of these mechanisms with the known information plant systems seems less plausible. However, from our results, it appears that there are specific changes occurring at molecular level in the seed systems (Figs 7 and 8). The non-enrichment of plant hormone pathway in fast freezing treatment observed by RNA-seq was probably a consequence of quick response of seeds to these events. Thus, most of the molecular changes occurred at higher sub-zero temperatures in fast cooled seeds. Based on this evidence, we suggest that a sudden cold shock stimulates all these changes when the temperature around seeds is rapidly reduced. By contrast, seeds do not undergo any changes at higher sub-zero temperature when the drop in temperature was slow at a more ecologically meaningful rate, presumably sensing the temperature change is a temporary stress. However, when the temperature was reduced to critical levels of −15 °C, a sudden burst of changes occurs (Figs 7 and 8), which contributes to seed survival at the slower cooling rate. Thus, similar to the physiological mechanisms, seeds have evolved with specific biochemical and molecular mechanisms to protect freezing stress that are specifically affected by cooling rate. Nevertheless, the significance of differential timing for regulation of above factors at different cooling rates demands special emphasis in the future studies.

## Methods

### Seed materials and germination

Seeds of *Lactuca sativa* cv. Iceberg were purchased from a retail outlet and stored at 15% RH and 15 °C prior to experimentation. The initial germination level was determined by sowing four replicates of 25 seeds on 1% agar-water in Petri dishes. The Petri dishes were incubated at 21 ± 1 °C constant temperature with 16/8 h light/dark regime with a light intensity of c. 30 μmol m^−2^ s^−1^. The seeds were deemed to have germinated when the radicle had emerged by 2 mm or more.

### Seed imbibition and moisture content

Ten replicates of 25 seeds were imbibed on 1% agar-water in Petri dishes one Petri dish was sampled every 8 hours for moisture content determination for 80 h. Moisture content was determined gravimetrically by oven drying at 103 °C for 17 h and expressed on a fresh weight basis throughout. The time at which the first germination took place was also recorded. Whenever, imbibed seeds were used in the experiments, the seeds took water for 12 hours (see results).

### Freezing tests

Fully imbibed, but non-germinated seeds (see Fig. 1) were surface dried by placing them between soft tissue pads. These seeds were loaded in cryovials and cooled in a programmable freezer at 60 °C or 3 °C h^−1^ to the terminal temperature of −20 °C. Viability assessments were made by sampling three replicates of 25 seeds at each 5 °C interval and germinating them under conditions described above.

### The relative electrolyte leakage (REL) assay

REL analysis was conducted in imbibed seeds with both non-freezing treatments and cooled to −5, −10, −15 and −20 °C at fast and slower rates using a method described by Chowhan *et al*.^54^. Briefly, 0.5 g seeds were added into the 50 ml-tubes with 5 mL deionized water, then mixed thoroughly by placing the tubes in a shaker (Kylin-Bell Lab Instruments Co. Ltd., Jiangsu, China) for 2 h before measuring the electrolyte leakage (EL) using a conductivity meter (Cold-Parmer, USA). EL was also measured after boiling the tubes with contents for 30 min. REL was expressed as the ratio of initial to final conductivity. De-ionised water was used as a control. The experiment is replicated twice and the values are presented as average.

### Detection of soluble sugar changes by HPLC

Previous studies on lettuce, and data collected in the present investigation (see results) indicate that supercooling ability is generally lost in the region of −18 °C; therefore, any molecular change contributing to the survival of seeds is expected to occur at temperatures above −20 °C. Consequently, we hypothesized cooling seeds to −20 °C could serve as an appropriate threshold for studying the importance of soluble sugar content. To determine the sugar level changes, imbibed seeds were cooled separately at 60 °C or 3 °C h^−1^ to −20 °C in a programmable freezer. These seeds were ground in a mortar and pestle in the presence of liquid nitrogen. The powder was homogenized by adding an equal volume of 80% ethanol and incubated at 50 °C for 20 min. The homogenate was then centrifuged for 30 min at 3000 rpm and the supernatant was collected and the remaining pellets were washed with 80% ethanol and re-centrifuged. This process was repeated thrice and all the supernatants collected were mixed. The ethanol in the supernatant was removed with a rotary vacuum evaporator at 50 °C. The remaining liquid was filtered with a 0.45 uM microporous filter membrane (Millipore, USA). The filtrate was diluted 10 fold by adding ddH_2_O and analyzed by HPLC with a Waters Associates Liquid Chromatograph (Milford, MA, USA), equipped with an isocratic1525 pump, U6 K injector. Sucrose was detected with a 2414 differential refractometer. The immobile phase was A P S- H Y P E R S IL (4.6 mm × 250 mm) and the mobile phase was acetonitrile: water (80:20) at a flow rate of 0.8 ml/min at room temperature. The volume of injection was 10 uL. The standard samples were prepared as follows: the sucrose samples were dried at 105 °C for 5 hr and 0.5 g sample was dissolved in water and diluted to 1, 2, 5, 10 mg/ml, respectively and filtered with a 0.45 uM membrane (Millipore, USA).

### Superoxide Dismutase (SOD) assay

Total SOD activity was measured following the method described by according to Beyer and Fridovich^55^. The total SOD activity was detected in imbibed seeds with non-freezing treatment (control) and imbibed seeds cooled to −20 °C under both the cooling rates. The total SOD activity was assayed photochemically based on the inhibition of NBT reduction by superoxide radicals. One unit of SOD was defined as the amount of enzyme required to inhibit the reduction rate of NBT by 50% at 25 °C.

### Free proline assay

Free proline was extracted using the method developed by Bates *et al*.^56^. Imbibed seeds with non-freezing treatment (using as control) and imbibed seeds cooled to −20 °C under both the cooling rates were ground and mixed with 5 ml of 3% aqueous sulfosalicyclic acid. The mixture was boiled for 10 min and centrifuged at 12000 rpm for 10 min. The discarded supernatant was subsequently mixed with 2 ml acid-ninhydrin and 2 ml glacial acetic acid. After boiling for 10 min, the reaction mixture was extracted with 4 ml toluene by centrifugation for 5 min at 3000 rpm. Then the absorbance was measured at 515 nm using NANO DROP2000 (Thermo Fisher Scientific, Waltham, MA USA). Standard curves were made using 2 ml standard solutions of proline with different concentration, prior to sample measurement.

### ABA and IAA content assay

ABA and IAA was extracted by modified method of the previous report^57^. Briefly, two grams of seeds in fresh weight were grounded into powder. Add 20 ml 80% methanol and shaked overnight at 4 °C. Then the extracting solution was evaporated to a volume of 4 ml and extracted by chloroform and ethyl acetate seperately. The extracting solution was evaporated to be dry and the extracts was dilluted by methanol. ABA was detected by HPLC and IAA was detected by LC-MS based on the previous method^57^.

### Determination of molecular changes in freezing seeds using RNA-seq analysis

To unravel the molecular changes occurring in seeds during low temperature, RNA-seq analysis was performed in BGI company (Shenzhen, China) using imbibed seeds (control) and after cooling seeds to −20 °C at 60 °C or 3 °C h^−1^. For RNA extraction, seeds cooled to −20 °C were ground using a mortar and pestle in the presence of liquid nitrogen. The samples were first treated with DNase I to degrade any possible DNA contamination and the mRNA was enriched by using oligo (dT) magnetic beads on the ice. The mRNA was then sliced into short fragments by mixing the sample with fragmentation buffer. Using the short fragments, we synthesized the first strand of cDNA by using random hexamer-primer. Later, Buffer, dNTPs, RNase H and DNA polymerase I were added to synthesize the second strands. The double strand cDNA was purified with magnetic beads. End reparation and 3′-end single nucleotide A (adenine) addition was then performed. Finally, sequencing adaptors were ligated to the fragments. The fragments were enriched by PCR amplification. During the quality control step, Agilent 2100 Bioanaylzer and ABI StepOnePlus Real-Time PCR System were used to qualify and quantify the sample library, which was sequenced via Illumina HiSeqTM 2000.

Data filtering was carried out to obtain high quality reads as the clean reads including removing reads with adaptor sequences, removing reads in which the percentage of unknown bases (N) was greater than 10%, and removing low quality reads. If the percentage of the low quality base (base with quality value ≤5) was greater than 50% in a read, we define this read as low quality. Clean reads were mapped to reference sequences and/or reference gene set using SOAPaligner/SOAP2^58^. No more than 2 mismatches were allowed in the alignment. The expression level for each gene was determined by the numbers of reads uniquely mapped to the specific gene and the total number of uniquely mapped reads in the sample. Briefly, the gene expression level was calculated by reads Per kb Million reads (RPKM) method.

The probability of gene A expressed equally between fast and slow cooled samples was calculated as equation (1):


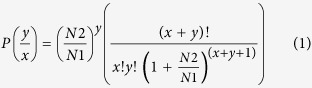


where, N1 is the number of total clean tags of the fast cooled sample, N2 is the number of total clean tags of slowly cooled sample, x is the number of tags held by gene A in slowly cooled sample, and y is the number of tags held by gene A in the slow cooled sample.

The calculated *P*-value corresponds to differential gene expression. False discovery rate (FDR) was used to determine the threshold of *P* value in multiple tests. We used “FDR ≤0.001 and the absolute value of log2Ratio ≥1” as the threshold to judge the significance of gene expression difference.

To classify the function of differentially expressed genes (DEGs), pathway enrichment analysis was performed by mapping the DEGs to KEGG database^59^, using the formula described by Hooper and Brok^60^. Briefly, this involves calculating gene numbers for every term, then using the hypergeometric test to find significantly enriched terms in DEGs. The following equation (2) was used to map the DEG’s to KEGG database:


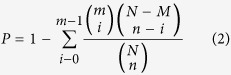


where N is the number of all genes with KEGG annotation, n is the number of DEGs in N, M is the number of all genes annotated to specific pathways, and m is the number of DEGs in M. The calculated *P* value was tested for statistical significance using Bonferroni correction, at *P* = 0.05.

### Verification by real time PCR

The RNAseq displayed a wave of changes occurring at −20 °C in both fast and slow cooled seeds (see results). To understand the temperature at which these changes occur in imbibed seeds at two different cooling rates, we verified the sequencing by extracting total RNA from imbibed seeds after cooling to −5, −10, −15, −20 °C at 60 °C or 3 °Ch^−1^. The samples were ground with a mortar and pestle in the presence of liquid nitrogen. The powdered seeds were treated with RNAiso Plus (TaKaRa) and cDNA was synthesized using reverse transcriptase (Prime Script RT reagent Kit with gDNA Eraser, Takara, Japan). Real time PCR was performed with PrimeScript™ RT reagent Kit and gDNA Eraser (Perfect Real Time) (Takara, Japan) by ABI7900 system (ABI, USA). The conditions were as follows: 1 cycle for degeneration at 94 °C (3 min), 40 cycles at 94 °C (20 s), 52 °C (20 s), 72 °C (20 s) for amplification and 1 cycle for elongation at 72 °C (7 min). Three replicates were carried out. The sequences of primers are listed in Supplementary Table 1.

### Statistical analysis

Percentage germination of seeds after cooling at 60 °C and 3 °Ch^−1^ to specific low temperature was compared using a *t-test*. The different groups of REL, SOD, proline and soluble sugars data of control, fast cooled and slow cooled seeds were tested for statistical significance by one-way ANOVA independently. We used LSD *post-hoc* test to find significant differences between groups.

## Additional Information

**How to cite this article**: Jaganathan, G. K. *et al*. Physiological Mechanisms Only Tell Half Story: Multiple Biological Processes are involved in Regulating Freezing Tolerance of Imbibed *Lactuca sativa* Seeds. *Sci. Rep.* **7**, 44166; doi: 10.1038/srep44166 (2017).

**Publisher's note:** Springer Nature remains neutral with regard to jurisdictional claims in published maps and institutional affiliations.

## Supplementary Material

Supplementary Table 1

## Figures and Tables

**Table 1:** Statistics of RNA-seq analysis.

Sample ID	Control	Fast cooling	Slow cooling
Total reads	12,413,353 (100.00%)	11,702,598 (100.00%)	11,527,110 (100.00%)
Total Base Pairs	608,254,297 (100.00%)	573,427,302 (100.00%)	564,828,390 (100.00%)
Total Mapped Reads	8,429,247 (67.90%)	7,730,392 (66.06%)	7,579,398 (65.75%)
Perfect Match	6,873,209 (55.37%)	6,296,355 (53.80%)	6,051,017 (52.49%)
<=2 bp Mismatch	1,556,038 (12.54%)	1,434,037 (12.25%)	1,528,381 (13.26%)
unique match	8,350,513 (67.27%)	7,651,551 (65.38%)	7,505,842 (65.11%)
Multi-position Match	78,734 (0.63%)	78,841 (0.67%)	73,556 (0.64%)
Total Unmapped Reads	3,984,106 (32.10%)	3,972,206 (33.94%)	3,947,712 (34.25%)

**Table 2:** The DEGs with up or down regulated between fast and slow cooling treatment compared with control.

GeneID	Blast nr	log2 Ratio(F/C)	Up-Down	FDR	log2 Ratio(S/C)	Up-Down	FDR
gi|90503171	lanC-like protein 2-like	−1.12	**Down**	3.45E-6	1.05	**Up**	5.43E−11
gi|22259961	leucine-rich repeat extensin-like protein 4-like	1.65	**Up**	4.53E-11	−2.01	**Down**	1.16E-4
gi|22250752	Clock regulated protein, TIME FOR COFFEE-like	1.49	**Up**	6.90E-57	−2.33	**Down**	2.18E-35
gi|22225830	−	1.44	**Up**	2.04E-17	−1.69	**Down**	6.08E-08
gi|22250698	−	1.41	**Up**	3.40E-28	−1.66	**Down**	5.37E-13
gi|22240524	−	1.36	**Up**	1.15E-13	−1.04	**Down**	0.000988
gi|22265151	unnamed protein product	1.33	**Up**	2.23E-18	−1.13	**Down**	9.17E-06
gi|22253147	DEAD-box ATP-dependent RNA helicase 42-like	1.32	**Up**	1.08E-28	−1.12	**Down**	1.35E-08
gi|22242137	unnamed protein product	1.16	**Up**	6.30E-13	−1.27	**Down**	1.14E-06
gi|22254644	glutamate synthase, putative (NADH dependent)	1.10	**Up**	4.62E-08	−1.34	**Down**	3.78E-05
gi|90517427	Unnamed protein	1.06	**Up**	9.68E-31	−1.40	**Down**	7.55E-22
gi|83979007	glutamate synthase, putative	1.04	**Up**	5.75E-18	−1.03	**Down**	2.19E-08

**Table 3:** The significantly enriched pathways in fast and slow cooling treatment.

Types	Pathways	^a^P-value(fast/slow)	^b^P-value (control/fast)	^c^P-value/(control/slow)	ID
I	RNA transport	2.95e–05	—	39 (0.38%)/0.001	ko03013
	Spliceosome	29 (3.68%)/0.001	—	32 (3.12%)/0.008	ko03040
	**Plant hormone signal transduction**	44 (5.58%)/0.001	—	55 (5.36%) 7.79–e4	ko04075
	Endocytosis	15 (1.9%)/0.032	—	18 (1.75%)/0.040	ko04144
	Pyrimidine metabolism	16 (2.03%)/0.041	—	20 (1.95%)/0.035	ko00240
	Nucleotide excision repair	8 (1.01%)/0.055	—	14 (1.36%)/0.001	Ko03420
II	Homologous recombination	6 (0.76%)/0.006	6 (0.66%)/0.011	6 (0.58%)/0.019	ko03440
	DNA replication	7 (0.89%)/0.013	7 (0.77%)/0.026	14 (1.36%)/5.53e–6	ko03030
	**Ubiquitin mediated proteolysis**	18 (2.28%)/0.064	23 (2.54%)/0.014	29 (2.82%)/0.001	ko04120
III	Alanine, aspartate and glutamate metabolism	15 (1.9%)/0.004	14 (1.55%)/0.025	14 (1.36%)/0.061	ko00250
	Fatty acid elongation	6 (0.76%)/0.063	8 (0.88%)/0.016	—	ko00062
IV	Protein processing in endoplasmic iculum	—	41 (4.54%)/0.002	40 (3.89%)/0.021	ko04141
	Circadian rhythm - plant	—	12 (1.33%)/0.021	13 (1.27%)/0.024	ko04712

Note: ^a^Pathway enriched using DEGs between fast and slow cooling treatment; ^b^Pathway enriched using DEGs between control and fast cooling treatment; ^c^Pathway enriched using DEGs between control and slow cooling treatment. I. Significant in slow cooling treatment; II. More significant in slow cooling than in fast cooling; III. More significant in fast cooling treatment; IV. Significant in both cooling treatments and no significant difference between the two kinds of cooling rates. Note: −*P* > 0.05.

**Table 4:** The response of plant hormone pathway genes to fast and slow cooling.

Temperature		Fast cooling				Slow cooling				Response to cold	Expression correlation of detected genes in single pathway
Pathways Genes		−5 °C	−10 °C	−15 °C	−20 °C	−5 °C	−10 °C	−15 °C	−20 °C^*^		
Auxin related	ARF1	0	+++^*^	+	−	+	0	0	+^*^	Positive	Antagonistic
	TIR1	0	—*	0	0	0	0	0	—*	Negative	
Ethylene related	EBF	0	—*	0	−	0	0	+	—*	Negative	Synergestic
	EIN2	0	—^*^	0	0	0	0	+	—*	Negative	
ABA related	ABF	+++^*^	++^*^	+	0	0	0	0	++*	Positive	—
Ubiquitin related	Skp1	0	0	+++^*^	−	0	0	+	+++*	Positive	Synergestic
	F-box	0	+++^*^	0	0	0	0	0	+++*	Positive	

Note: *indicated that the response occurred at the early stage of fast cooling or later stage of slow cooling. “+” indicated up-regulation and “−” indicated down-regulation. The number of “+” or “− ”indicated the significance of the differential expression. If the number was 1, 0.001 < *P* < 0.05. If the number was 2, 0.0001 < *P* < 0.001. If the number was 3, *P* < 0.0001, “0” indicated that *P* > 0.05.
