# Supplementary material for: Physiological Mechanisms Only Tell Half Story: Multiple Biological Processes are involved in Regulating Freezing Tolerance of Imbibed Lactuca sativa Seeds
Source: Sci Rep. 2017 Mar 13;7:44166. doi: 10.1038/srep44166 (PMC5347015; doi:10.1038/srep44166)
Supplement: Supplementary Table 1 [file srep44166-s1.doc]

**Title Page**

**Physiological Mechanisms Only Tell Half Story: Multiple Biological Processes are involved in Regulating Freezing Tolerance of Imbibed *Lactuca sativa* Seeds**

Ganesh K. Jaganathan^¶^，Yingying Han^¶^, Weijie Li, Danping Song, Xiaoyan Song, Mengqi Shen, Qiang Zhou, Chenxue Zhang, Baolin Liu*

Institute of Biothermal Science and Technology, University of Shanghai for Science and technology, 516 Jungong Road, Shanghai 200093, China.

Supplementary Table 1 The primers of real time PCR

| Primer name | Sequences(5’ to 3’) |
| --- | --- |
| ARF1-F | TAACAATAACAATAGCAACGAAC |
| ARF1-R | TTGAAGGCGATGTGGAAC |
| TIR1-F | GGTTGTTTGGGTTGTGAG |
| TIR1-R | GGATGTCTGCTGAGTAGG |
| EIN2-F | CGAAGGCAACACCACATC |
| EIN2-R | GATTGAAGACGGAGGCTATG |
| EBF-F | TCTCTTACCCTATTGAATCTCTCC |
| EBF-R | GCCTTCGTTCGTGATGTTTG |
| ABF-F | CACGAGAAGAAGAAGAAGAATCC |
| ABF-R | AACACGCTATTGGCTTGG |
| Skp1-F | GAGGCGATTAAGCAAATG |
| Skp1-R | ATGAACATCCACGAACTG |
| F-BOX-F | CCTCACCTCAAGTCCTTCAAG |
| F-BOX-R | CTGTTAAGAATGGCTTCAAGTCC |
| ACTIN -F | CTGGTGTGATGGTAGGTATGG |
| ACTIN -R | CTCGTTGTAGAAAGTGTGATGC |
